# Seizures, Vitamin D Deficiency, and Severe Hypophosphatemia: The Unique Presentation of a SARS-CoV-2 Case

**DOI:** 10.7759/cureus.33303

**Published:** 2023-01-03

**Authors:** Benson Yeh, Christopher Bell, Christopher Anderson, Mack Sheraton

**Affiliations:** 1 Emergency Department, Trinity West Medical Center, Steubenville, USA; 2 Emergency Department, West Virginia School of Osteopathic Medicine, Lewisburg, USA; 3 Emergency Medicine, Emory University School of Medicine, Atlanta, USA

**Keywords:** sars-cov-2 infection, neurological manifestations in covid-19, vitamin-d deficiency, hypophosphatemia, recurrent seizures

## Abstract

Severe acute respiratory syndrome coronavirus 2 (SARS-CoV-2) is a virus that belongs to the species severe acute respiratory syndrome-related coronavirus (SARSr-CoV), which is related to the SARS-CoV-1 virus that caused the 2002-2004 SARS outbreak. SARS-CoV-2 causes coronavirus disease 2019 (COVID-19). It has been associated with electrolyte abnormalities. In this report, we discuss the case of a SARS-CoV-2-infected person presenting with recurrent seizure episodes resulting from hypophosphatemia.

A 52-year-old male patient with questionable prior seizure history presented to the emergency department (ED) twice within eight days with recurring seizure episodes. While the physical examination at the first presentation was significant for a head laceration with post-ictal confusion, that at the second presentation was only significant for post-ictal confusion. Laboratory examination at the first visit revealed SARS-CoV-2 positivity, hypokalemia, hypophosphatemia, and low vitamin D levels. On the second visit, the patient was again found to have hypophosphatemia. CT of the head and the cervical spine, as well as radiographs of the chest done on the first visit, were all normal. On his first visit, the patient’s electrolyte abnormalities were corrected, and he was discharged with antiepileptic medications after 24 hours of observation and consultation with neurology. However, his vitamin D levels, the results of which came back only after his first discharge, were corrected only during his second visit. This time, he was discharged from the ED and had an effective resolution of symptoms. SARS-CoV-2 infections can result in vitamin D deficiency and hypophosphatemia, resulting in seizures, and hence should be treated with both replacement therapies and antiepileptic medications. This case also highlights the importance of obtaining phosphorus and vitamin D levels in SARS-CoV-2-infected patients with seizures.

## Introduction

Severe acute respiratory syndrome coronavirus 2 (SARS-CoV-2), which causes coronavirus disease 2019 (COVID-19), has been an intense topic of discussion, debate, and research since the initial cases were reported in late 2019. As of December 30, 2022, the World Health Organization (WHO) has reported a total of more than 660 million cases of COVID-19 and more than 6.6 million deaths worldwide. As reported by WHO in 2022, multiple variants of this virus have continued to evolve since the onset of the pandemic, with a gradual decrease in the severity of symptoms and presentations. However, their incubation periods have also decreased. Variants like Omicron with decreased incubation periods could spread more rapidly, which has resulted in about 444,072 cases as of today. This virus continues to place a heavy burden on our communities and healthcare systems. Common presentations of SARS-CoV-2 infection have been extremely varied, ranging from common flu-like symptoms to hypoxia, respiratory failure, and multiorgan failure. Incidences of neurological symptoms such as headaches, dizziness, encephalitis, and seizures have also been described [1,2]. A case series involving seven SARS-CoV-2 patients associated with seizures did not explore hypophosphatemia as a possible etiology [3].

Proposed causal mechanisms of severe CNS manifestations of SARS-CoV-2 typically involve the entry of the virus into the CNS and the eventual development of meningitis or encephalitis [4]. Angiotensin-converting enzyme 2 (ACE2) receptors, present in great concentrations in neuronal and glial cells, have often been hypothesized to be a probable pathway for viral access to the neuronal cells along with direct access through the olfactory bulb [5]. Metabolic mechanisms, however, are less commonly described. Malieckal et al. have reported a wide range of derangements in the electrolyte levels obtained from 11,635 SARS-CoV-2-affected subjects. However, they did not propose any mechanisms for such abnormalities. Hyponatremia, hypochloremia, and hypocalcemia were frequently detected. Still, hypophosphatemia was not well reported because, in 39.8% of cases, the phosphorus level was never tested [6]. Multiple case reports have associated hypophosphatemia with seizures, although the causality has not been firmly established [7-9]. Vitamin D deficiency causing severe hypophosphatemia was proposed as a mechanism for the precipitation of seizures in patients with SARS-CoV-2 [10]. In a multicenter retrospective study investigating seizure risk factors in 304 SARS-CoV-2 patients, one patient was found to have hypocalcemia-associated myoclonus. However, there was no mention of associated phosphorus levels [11]. Some authors have proposed GABA-mediated mechanisms for seizures, while others have implicated cytokines [12,13]. In a narrative review, Kuroda has observed that prospective trials on epilepsy and SARS-CoV-2 have been scarce [14]. We report the case of a patient who presented with the sole symptom of recurrent seizures and had associated severe hypophosphatemia and vitamin D deficiency.

## Case presentation

**Figure 1:**
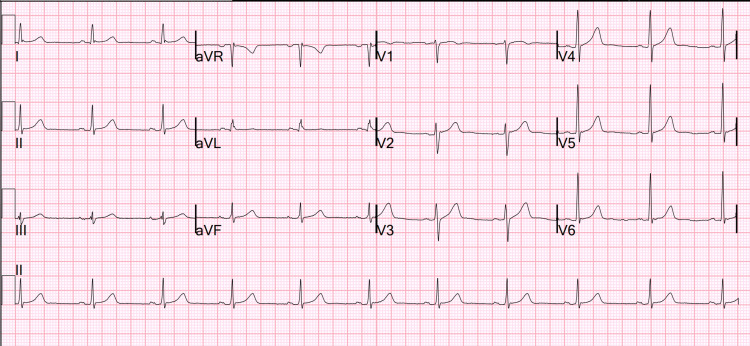
Electrocardiogram at presentation

A 52-year-old male patient presented to the emergency department (ED) following a witnessed seizure approximately 30 minutes prior to the arrival. Emergency medical services (EMS) reported that the patient had been at his workplace when he was seen by coworkers to stiffen and fall. He had struck his head on a sawhorse and sustained a laceration. This had been followed by convulsions, which had lasted for approximately five minutes. Convulsions had been tonic-clonic involving his whole body. EMS arrived to find the patient postictal and bleeding from a posterior head laceration. A pressure dressing had been applied and the patient had been transported to our ED. There had been no other seizure activity during transport. History obtained from the patient, his family, and his electronic medical records indicated that the patient had a past history of two seizure episodes - one in 2017 and again in 2019 - in the last five years due to an unknown etiology. A prior EEG done in 2017 was normal and he had never been put on any antiepileptic medications. He had been in his usual state of good health just prior to this seizure episode. He denied any headache, dizziness, fatigue, fever, chills, sore throat, nasal congestion, nausea, vomiting, abdominal pain, weakness, numbness, tingling, chest pain, shortness of breath, recent illness, or sick contacts. He worked in a steel mill, primarily involved in moving inventory, and had no exposure to welding or other chemical toxins. He did smoke but denied consuming alcohol or any other illegal substances. He lived with his wife and son and had a family history positive for hypertension, congestive heart failure, and coronary artery disease in his father and grandfather. The patient was not vaccinated against either influenza or COVID-19. Initial EKG done at triage showed normal sinus rhythm (Figure 1). Vitals were normal at presentation and physical examination was significant for a 2-cm superficial occipital scalp laceration and postictal confusion.

**Figure 2:**
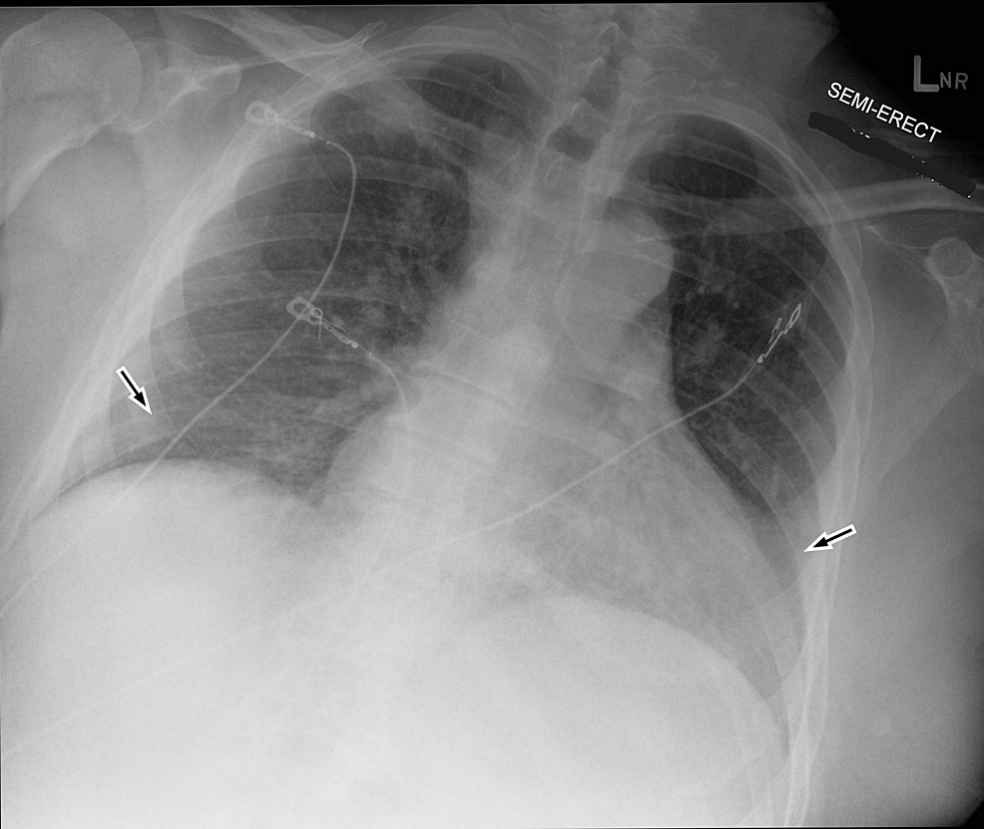
Chest radiograph during the first visit Black arrows indicate bibasilar atelectasis

A comprehensive workup with a complete blood count, complete metabolic panel, phosphorus, magnesium, urine drug screen, urinalysis, alcohol level, and a viral panel was ordered. The only abnormalities detected on serology were hypokalemia of 3.1 mmol/L (normal range: 3.6-5.2 mmol/L), hypophosphatemia of 1.0 mg/dL (normal range: 2.6-4.5 mg/dL), and positivity for SARS-CoV-2 by PCR (Table 1). Given these abnormal electrolytes, subsequently, intact parathyroid hormone, serum vitamin D2 and D3, and a full respiratory pathogen panel were ordered. Imaging studies, which included a CT scan of the head (Video 1) and the cervical spine (Video 2) and a radiograph of the chest (Figure 2), were all within normal limits. The patient was given 250-cc normal saline bolus with 15-mmol potassium phosphate rider. The laceration was repaired with staples, and the patient was admitted for observation and neurology consultation. A 16-channel EEG done later in the day showed bursts of high amplitude slowing suggestive of a postictal state. The patient was repleted with phosphorus and discharged at a level of 2.8. He was discharged home on 500 mg of levetiracetam twice daily the next day. Since vitamin D2/D3 levels were send-out tests, their results had not returned at the time of discharge.

**Table 1:** Summary of laboratory test results drawn on the first presentation WBC: white blood cell; Hb: hemoglobin; Hct: hematocrit; MCV: mean corpuscular volume; MCH: mean corpuscular hemoglobin; MCHC: mean corpuscular hemoglobin concentration; RDW: red cell distribution width; SARS-CoV-2: severe acute respiratory syndrome coronavirus 2; RT-PCR: reverse-transcription polymerase chain reaction; PT: prothrombin time; INR: international normalized ratio; BUN: blood urea nitrogen; GFR: glomerular filtration rate; AST: aspartate transaminase; ALT: alanine transaminase; ALP: alkaline phosphatase; PTH: parathyroid hormone

Test	Results	Reference
Hematology		
WBC	9	4.5–11 x 10^3^uL
Hb	16.5	13.0–16.0 g/dl
Hct	47.2	41–53%
MCV	94.7	78–98 fl
MCH	33.0	26.0–34.0 pg
MCHC	34.8	31.0–37.0 g/dl
RDW	13.1	11.5–14.5%
Plt count	208	150–350 x 10^3^/uL
Microbiology		
SARS-CoV-2 (PCR)	Detected	Not detected
Influenza A (RT-PCR)	Not detected	Not detected
Influenza B (RT-PCR)	Not detected	Not detected
RSV (PCR)	Not detected	Not detected
Respiratory viral panel (PCR)	Not detected	Not detected
Bordetella pertussis	Neg	Neg
Toxicology		
Urine drug screen	None detected	None detected
Urine specific gravity	1.013	1.002–1.03
Coagulation		
PT	10.9	9.6–11.2 seconds
INR	1.1	
APTT	27	22–23 seconds
Chemistry		
Sodium	137	136–145 mmol/L
Potassium	3.1	3.5–5.1 mmol/L
Chloride	108	98–107 mmol/L
Bicarbonate	22	21–32 mmol/L
Anion gap	10.1	0–16
BUN	9	7–18 mg/dL
Creatinine	1.23	0.70–1.30 mg/dL
Estimated GFR	>60	>60
Glucose	139	70–110 mg/dL
Calcium	8.6	8.5–10.1 mg/dL
Phosphorus	1.0	2.5–4.9 mg/dL
Magnesium	2.2	1.6–2.6 mg/dL
Total bilirubin	0.60	0.0–1.0 mg/dL
AST	26	15–37 U/L
ALT	26	13–61 U/L
ALP	75	45–117 U/L
Albumin	3.5	3.4–5.0 g/dL
Send-outs received on day 4		
Vitamin D2 and D3 total	19.3	30.0–80.0 ng/ml
Vitamin D3	19.3	
Vitamin D2	<1	
Intact PTH	32	15–65 pg/ml
Calcium for PTH	9.0	8.6–10.0 mg/dl

**Video 1:** Head CT performed during the first visit The procedure showed normal results CT: computed tomography

**Video 2:** Cervical spine CT The CT was negative for anything acute like vertebral fracture or malalignment CT: computed tomography

The patient returned to the ED eight days later after another witnessed five-minute seizure episode at home. Vitals were normal and physical examination this time was only significant for resultant postictal confusion. A metabolic panel obtained upon ED arrival again demonstrated hypophosphatemia of 1.9 mg/dL (normal range: 2.6-4.5 mg/dL) (Table 2). After the initial postictal period, the patient showed medication compliance. He was again treated with potassium phosphate and given 1000 mg of oral levetiracetam. By this time, his prior labs had returned, showing low vitamin D2/3 levels for which he was placed on a vitamin D replacement regimen. A Keppra level obtained was also found to be borderline therapeutic, indicating compliance with therapy. This time, he was discharged home from the ED after consultation with the neurology team. His Keppra dosing was doubled at discharge and he was referred to endocrinology for further follow-up for the hypophosphatemia. Subsequently, he had a sustained resolution of his recurrent seizure episodes. Table 3 shows a comprehensive timeline of events.

**Table 2:** Summary of laboratory test results on the second visit WBC: white blood cell; Hb: hemoglobin; Hct: hematocrit; MCV: mean corpuscular volume; MCH: mean corpuscular hemoglobin; MCHC: mean corpuscular hemoglobin concentration; RDW: red cell distribution width; BUN: blood urea nitrogen; GFR: glomerular filtration rate; AST: aspartate transaminase; ALT: alanine transaminase; ALP: alkaline phosphatase

Test	Results	Reference
Hematology		
WBC	4.5	4.5–11 x 10^3^uL
Hb	17	13.0–16.0 g/dl
Hct	49.7	41–53%
MCV	96	78–98 fl
MCH	32.8	26.0–34.0 pg
MCHC	34.1	31.0–37.0 g/dl
RDW	13.7	11.5–14.5%
Plt count	200	150–350 x 10^3^/uL
Toxicology		
Levetiracetam level	11	10–40 ug/ml
Chemistry		
Sodium	139	136–145 mmol/L
Potassium	3.7	3.5–5.1 mmol/L
Chloride	109	98–107 mmol/L
Bicarbonate	22	21–32 mmol/L
Anion gap	11.7	0–16
BUN	9	7–18 mg/dL
Creatinine	1.28	0.70–1.30 mg/dL
Estimated GFR	59	>60
Glucose	139	70–110 mg/dL
Calcium	8.9	8.5–10.1 mg/dL
Phosphorus	1.9	2.5–4.9 mg/dL
Magnesium	2.3	1.6–2.6 mg/dL
Total bilirubin	0.30	0.0–1.0 mg/dL
AST	14	15–37 U/L
ALT	24	13–61 U/L
ALP	84	45–117 U/L
Albumin	3.7	3.4–5.0 g/dL

**Table 3:** Timeline of events ED: emergency department; EMS: emergency medical services; EEG: electroencephalogram; AED: antiepileptic drug

Timeline	Event
Day 1	
0933	Witnessed seizures at the workplace
1010	Presents to the ED with EMS
1036	Workup initiated in the ED
1315	Neurology consultation received
1540	Admission for observation
1650	EEG confirmed post-ictal state
Day 2	
0830	Discharged with AED and follow up
Day 4	
1045	Deficient vitamin D2/D3 levels reported
Day 8	
1040	Seizures witnessed at home
1100	Presents to ED with EMS
1115	ED workup initiated
1340	Neurology consultation received
1500	Discharged with outpatient follow-up

A review of records related to subsequent outpatient workups revealed evaluations with the neurologist and endocrinologist. Endocrinology workup included genetic testing and interval measurements of serum phosphate, calcium, alkaline phosphatase, parathyroid hormone, 25-hydroxyvitamin D, 1,25-hydroxyvitamin D, fibroblast growth factor 23, urine calcium, and the ratio of tubular maximum reabsorption rate of phosphate-to-glomerular filtration rate (TmP/GFR). All of these values were normal, and the patient was not recommended for subsequent follow-up. Neurology workup included MRI to look into structural causes, which came back normal.

## Discussion

Phosphorus has been shown to have multiple implications in the cells of the CNS and the body as a whole. Phosphorus is an integral part of adenosine triphosphate (ATP), adenosine monophosphate (AMP), and phospholipid membranes in cell membranes and organelles, making it a crucial piece in the body’s metabolism and function [15]. Phosphorus has been reported to be found depleted in several studies relating to tonic-clonic seizures, and some hypothesize that it may be a nonspecific marker for seizures [16]. It has also been shown that low phosphorus levels can be detrimental to the energy demands required by the brain, leading to multiple neurological symptoms such as numbness, weakness, seizures, and coma in patients with exceptionally low phosphorus levels, i.e., 1.0 mg/dL or less [10].

The SARS-CoV-2 infection has been shown to cause a systemic immune response leading to a massive release of cytokines, inflammatory mediators, and immune cells in response to the infection. This results in the upregulation of multiple immune system pathways in an attempt to clear the virus from the body. These processes consume ATP as the inflammatory response and production of immune cells need energy. Concomitantly, depletion of inorganic phosphates occurs, which has been demonstrated in SARS-CoV-2 infections [17,18]. Interestingly, vitamin D acts as an immune modulator, and its low levels are associated with cytokine release, with hypophosphatemia reported as a harbinger of severe disease [10]. Our patient had serum vitamin D2 and D3 levels drawn on the date of the first presentation. A combined deficiency at the level of 19.3 (30-80 ng/mL) was reported only after the patient’s discharge. Therefore, supplementation of phosphate and vitamin D should be considered in deficient patients at risk for severe COVID-19 disease outcomes [10].

We postulate that low vitamin D levels concurrent with SARS-CoV-2 infection led to severe hypophosphatemia of 1.0 mg/dL (normal range: 2.6-4.5 mg/dL) in our patient, resulting in seizure episodes. On the patient’s second presentation for recurring seizures, he again demonstrated moderate hypophosphatemia despite compliance with the anti-seizure medication levetiracetam. Continued low levels of phosphorus in this patient support our observations of seizures secondary to SARS-CoV-2 infection, as the one-time phosphorus replacement and antiepileptic medications made no change in presentation. We hypothesize that the lower phosphorus levels secondary to a SARS-CoV-2 infection in our patient led to a decrease in seizure threshold, thereby resulting in his seizures. Lastly, we highlight the importance of ensuring follow-up for send-out tests drawn in the ED. If the results related to deficient vitamin D levels had reached the patient's primary care physician earlier and if he had promptly acted on it, we do not think the recurrence would have occurred.

## Conclusions

To our knowledge, this case report is the first to describe seizures resulting from severe hypophosphatemia and low vitamin D levels as an initial presentation of SARS-CoV-2 infection. Seizures are an uncommon presentation of SARS-CoV-2 infections. The epileptic mechanism in SARS-CoV-2 still remains unclear. However, it is well known that vitamin D modulates the severity of SARS-CoV-2 systemic inflammatory response. Additionally, low vitamin D plays a significant role in calcium and phosphorus metabolism. Depletion of vitamin D results in the upregulation of inflammatory markers and immune cells, which places an increased demand on the body's energy stores. Inorganic phosphorus is consumed, which can result in a severe deficiency of this metabolite.

We also note that phosphorus levels are frequently not checked in patients who present with seizures and SARS-CoV-2. We recommend early testing to determine whether COVID-19 patients are experiencing seizures due to transient hypophosphatemia in the setting of vitamin D deficiency. Lastly, it is evident that fluctuations of phosphorus levels in SARS-CoV-2 are not very well studied. We recommend future research to delineate the exact mechanism behind the association of phosphorus, vitamin D, and seizures with COVID-19 infection.
